# Comparison of heart failure risk assessment tools among cancer survivors

**DOI:** 10.1186/s40959-024-00267-5

**Published:** 2024-10-11

**Authors:** Cheng Hwee Soh, Thomas H. Marwick

**Affiliations:** 1 Imaging Research Laboratory, Baker Heart and Diabetes Institute, Melbourne, Australia; 2 Baker Department of Cardiometabolic Health, University of Melbourne, PO Box 6492, Melbourne, Victoria 3004 Australia; 3 Menzies Institute for Medical Research, Hobart, Australia; 4 Baker Heart and Diabetes Institute, PO Box 6492, Melbourne, VIC 3004 Australia

**Keywords:** Heart failure, Prognosis, Cancer, Risk assessment

## Abstract

**Background:**

Cancer survivors have an increased risk of incident heart failure (HF) attributable to shared risk factors and cancer treatment-induced cardiac dysfunction. Selection for HF screening depends on risk assessment, but the optimal means of assessing risk is undefined. We undertook a comparison of HF risk calculators among survivors.

**Methods:**

In this study from the UK Biobank, cancer and HF diagnoses were determined based on the International Classification of Diseases (ICD)-10 code and non-cancer participants were included as controls. Participants’ risk of incident HF was determined using the Heart Failure Association-International Cardio-oncology Society (HFA-ICOS), the Atherosclerosis Risk in Communities (ARIC-HF) and the Pooled Cohort Equations to Prevent Heart Failure (PCP-HF). The predictive performances of each were compared using the area under the curve (AUC).

**Results:**

After propensity matching with age and sex, 9,232 survivors from breast cancer or lymphoma (mean age 59.9 years, 87.8% female), and 23,800 survivors from other cancer types (mean age 59.1 years, 85.8% female) were included in the analysis. The discriminative value for HFA-ICOS (AUC 0.753 [95%CI: 0.739–0.766]) and ARIC-HF (0.757 [95%CI: 0.744–0.770]) were similar, and superior to PCP-HF (0.717 [95%CI: 0.702–0.732]). The overall performance for each risk score was better among participants in other cancer types than those with breast cancer and lymphoma.

**Conclusions:**

HFA-ICOS and ARIC-HF outperformed the PCP-HF among cancer- and non-cancer cohort, although all showed modest discrimination for incident HF to be applied to clinical practice. A cancer-specific HF prediction tool could facilitate HF prevention among survivors.

**Supplementary Information:**

The online version contains supplementary material available at 10.1186/s40959-024-00267-5.

## Introduction

The risk of heart failure (HF) is increased in cancer survivors. As HF is a leading cause of hospitalization with high mortality rate [1], HF prevention in survivors would not only provide significant individual clinical value, but also reduce the overall burden on the healthcare system [2]. 

The causes of HF in survivors is well-understood, including shared risk factors between cancer and HF [3], and potentially cardiotoxic cancer treatment such as radio- and chemotherapy [4]. Various risk calculators seek to integrate these into an assessment of overall risk, but the optimal approach to HF risk prediction in survivors is unclear. The only adult cancer-specific HF risk prediction score is the Heart Failure Association-International Cardio-Oncology Society (HFA-ICOS) score. It accounts for cancer-related factors but was developed for risk assessment at the time of chemotherapy, hence its feasibility and prognosis value for HF incidence in the application within the primary care setting remained uncertain [5]. Extending the search of HF risk prediction tools to non-cancer cohort, incident HF has been predicted in the general population using the Atherosclerosis Risk in Communities (ARIC-HF) [6] and modified Pooled Cohort Equations to Prevent Heart Failure (PCP-HF) [7] scores, but their discriminative performance among survivors is unclear. Hence, in this study, we sought to compare the performance of current these HF risk assessment tools for prediction of incident HF among survivors. Based on the increased risk of HF with breast cancer and lymphoma, we also aimed to investigate if the performance differed between cancer types.

## Methods

### Study design and setting

This analysis used the UK-Biobank - a large population-based, prospective cohort study with roughly 500,000 participants aged 35 years or above [8]. Participants were recruited from 2006 to 2010 and are still being followed up to date. Available data include sociodemographic characteristics, lifestyle, physical examination, diseases and symptoms, pathology results and self-report questionnaires. The present analysis was conducted under the application ID 55,469 for analysis of the UK-Biobank data, approved by the North West – Haydock Research Ethics Committee (16/NW/0274). The study followed the guidelines outlined in the Declaration of Helsinki, and written informed consent was provided by all participants.

### Cancer diagnosis

Cancer diagnoses were determined based on the tenth revision of the International Classification of Diseases (ICD-10) code, from C00 to C97 and D00 to D45 [9]. A sub-population was identified with breast cancer (C50) or lymphoma (C81-C96). Participants without a history of cancer diagnosis were included as controls for this study.

### CVD risk assessment tools

Cancer survivors were classified into low, medium, high and very high risk based on the HFA-ICOS score (Supplementary Table 1) [5]. However, due to the limited data on participants’ cardiac biomarkers and cancer treatment history, these parameters were not evaluated to ensure consistency in the risk assessments among all cancer survivors. The 10-year probability of incident HF was calculated using the ARIC-HF score [6] (Supplementary Table 2). The Modified Pooled Cohort Equations to Prevent Heart Failure (PCP-HF) was also calculated for all included participants [7] (Supplementary Table 3). Similar to ARIC-HF, PCP-HF were presented as the probability of an event in the next 10 years. The date of consent to the UK Biobank study was considered as the baseline, and all risk assessments were conducted using the data collected at that timepoint.

### Other clinical characteristics

Other clinical characteristics such as alcohol consumption (Field ID 20117), diastolic blood pressure (Field ID 4079), the International Physical Activity Questionnaire (IPAQ) activity level (Field ID 22032) and a self-reported overall health rating (Field ID 2178) were included in this analysis. Diagnoses of comorbidities such as hypercholesterolemia, chronic kidney disease, hypertension and obesity were also computed based on ICD-10 code. Hypertension could also be determined based on elevated blood pressure (systolic > 140mmHg or diastolic > 90mmHg) or use of blood pressure lowering medications. A 2 × 2 contingency table demonstrating the differences between hypertension diagnoses based on ICD10 code, elevated blood pressure and the use of blood pressure lowering medications were reported in Supplementary Table 4.

### HF incidence

Similar to cancer diagnoses, incident HF was determined based on a documentation of diagnosis in the ICD-10 code – I50. Date of HF diagnosis was also documented and the duration from date of consent to date of HF diagnosis was calculated. Participants who had a history of HF diagnosis prior to consent were excluded from the study.

*Statistical analysis.* A propensity matching was performed based on participants’ age and sex. The baseline characteristics for the participants were reported using descriptive statistics. All continuous variables were reported as means ± standard deviation (SD) The baseline characteristics were compared using t tests for parametric variables and chi-square tests for categorical variables. The cumulative incidence plots for incident HF were generated for the two subpopulations of cancer participants and controls. The cumulative incidence plots, stratified according to age-range were also produced.

To compare the performance of the CVD risk assessment tools in predicting incident HF, the area under the receiver operating characteristics (ROC) curve (AUC) was used and reported as AUC (95% Confidence Interval, CI). The AUC and the corresponding sensitivity and specificity for each of the tools were reported among all included participants, as well as the subpopulations of breast cancer and lymphoma survivors and other cancer types. With death being set as the competing risk, Fine and Gray competing risk regression analyses were also performed to evaluate the risk factors associated with HF incidence. The univariable competing risk regression model for each characteristic was reported with the effect size of subdistribution hazard ratio (SHR) and the 95% confidence interval (CI). Key risk factors were then selected via a stepwise backward regression approach, with only variables with p-value of less than 0.05 maintained in the model, to generate the multivariable regression model for HF incidence. A *p*-value of less than 0.05 was considered statistically significant. Participants with missing data were excluded from the analysis. All analyses were conducted in R (R Foundation for Statistical Computing).

### Patient and public involvement

Patients and/or the public were not involved in the design, or conduct, or reporting or dissemination plans of this research.

## Results

### Clinical characteristics

After excluding 2,644 participants with HF diagnosis prior to UK-Biobank consent, 43,720 participants with cancer were included in this study. A total of 440,813 non-cancer participants were also included as controls. Overall, 9,232 participants were diagnosed with breast cancer or lymphoma, with 34,488 participants with other cancer types (Supplementary Table 5). Propensity matching based on age and sex resulted in 9,232 breast cancer or lymphoma participants, 23,800 participants with other cancer types and 99,096 controls included in the analyses (Table 1). Participants with breast cancer or lymphoma had a significantly higher body mass index and blood pressure. The prevalence of chronic kidney disease was significantly higher among participants with breast cancer or lymphoma. The physical fitness and overall health rating among participants with breast cancer or lymphoma were also significantly lower (*p* = 0.001 and *p* < 0.001, respectively). There was no statistically significant difference in the ARIC-HF (1.01% vs. 0.98%, *p* = 0.127) score between the two groups, but the participants with breast cancer or lymphoma had significantly higher PCP-HF (2.47% vs. 2.36%, *p* < 0.001) scores than those with other cancer types. By contrast, the ARIC-HF and PCP-HF scores for non-cancer participants were 0.98 ± 1.64% and 2.33 ± 1.76% respectively.

**Table 1:** Characteristics of non-cancer participants and participants with cancer

	Non-cancer participants (n = 99,096)		Breast cancer and lymphoma (n = 9,232)		Other cancer types (n = 23,800)		p-value*
Age (y)	59.28 ± 7.51		59.86 ± 6.98		59.06 ± 7.69		0.584
Female, n (%)	85,548 (86.33)		8,103 (87.77)		20,413 (85.77)		0.063
Ethnicity, n (%)							**0.045**
White	93,930 (94.79)		8,930 (96.73)		23,160 (97.31)		
Asian	570 (0.58)		80 (0.87)		103 (0.43)		
Black	1,607 (1.62)		77 (0.83)		134 (0.56)		
Chinese	1,410 (1.42)		15 (0.16)		177 (0.74)		
Mixed	306 (0.31)		48 (0.52)		37 (0.16)		
Other	862 (0.87)		54 (0.58)		115 (0.48)		
Alcohol consumption, n (%)							0.234
Never	5,593 (5.64)		461 (4.99)		1,127 (4.74)		
Previous	3,630 (3.66)		385 (4.17)		987 (4.15)		
Current	89,649 (90.47)		8,364 (90.60)		21,645 (90.95)		
Smoking status, n (%)							**< 0.001**
Never	56,940 (57.46)		5,012 (54.29)		12,393 (52.07)		
Previous	33,265 (33.57)		3,391 (36.73)		8,698 (36.55)		
Current	8,364 (8.44)		787 (8.52)		2,577 (10.83)		
Body mass index (kg/m2)	27.27 ± 4.99		27.31 ± 4.90		27.05 ± 5.02		**< 0.001**
Total cholesterol (mmol/L)	5.87 ± 1.15		5.88 ± 1.20		5.87 ± 1.16		0.776
HDL-C (mmol/L)	1.56 ± 0.39		1.54 ± 0.40		1.56 ± 0.39		**< 0.001**
LDL-C (mmol/L)	3.64 ± 0.88		3.64 ± 0.91		3.63 ± 0.89		0.548
Blood pressure							
Systolic blood pressure (mmHg)	138.13 ± 19.33		138.27 ± 19.18		137.42 ± 19.34		**< 0.001**
Diastolic blood pressure (mmHg)	81.31 ± 10.00		81.49 ± 10.03		81.03 ± 9.99		**< 0.001**
Heart rate (bpm)	69.93 ± 10.91		72.11 ± 11.42		70.35 ± 11.05		**< 0.001**
Diabetes, n (%)	4,536 (4.58)		466 (5.05)		1,080 (4.54)		0.053
Coronary Heart Disease, n (%)	3,688 (3.72)		317 (3.43)		828 (3.48)		0.673
Hypertension, n (%)	673 (0.68)		56 (0.61)		156 (0.66)		0.673
Hypercholesterolaemia, n (%)	24 (0.02)		4 (0.04)		3 (0.01)		0.194
Chronic Kidney Disease, n (%)	279 (0.28)		62 (0.67)		103 (0.43)		**0.007**
Obesity, n (%)	179 (0.18)		10 (0.11)		44 (0.18)		0.163
Medications, n (%)							
Insulin	909 (0.92)		89 (0.96)		232 (0.97)		0.979
Blood pressure medication	21,211 (21.40)		2,038 (22.08)		5,005 (21.03)		**0.039**
Cholesterol lowering medication	16,407 (16.56)		1,433 (15.52)		3,907 (16.42)		**0.049**
IPAQ (%)							**0.001**
Low	14,011 (14.14)		1,479 (16.02)		3,524 (14.81)		
Moderate	32,854 (33.15)		3,065 (33.20)		7,756 (32.59)		
High	30,322 (30.60)		2,625 (28.43)		7,192 (30.22)		
Overall health rating, n (%)							**< 0.001**
Excellent	16,848 (17.00)		775 (8.39)		3,224 (13.55)		
Good	59,098 (59.64)		5,034 (54.53)		13,608 (57.18)		
Fair	19,027 (19.20)		2,585 (28.00)		5,507 (23.14)		
Poor	3,603 (3.64)		738 (7.99)		1,303 (5.47)		
ARIC-HF (%)	0.98 ± 1.64		1.01 ± 1.51		0.98 ± 1.63		0.127
PCP-HF (%)	2.33 ± 1.76		2.47 ± 1.89		2.36 ± 1.96		**< 0.001**
HFA-ICOS, n (%)							**0.003**
Low	-		5,273 (57.12)		13,847 (58.18)		
Medium	-		3,327 (36.04)		8,311 (34.92)		
High	-		528 (5.72)		1,370 (5.76)		
Very high	-		38 (0.41)		49 (0.21)		
Years since cancer diagnoses (years)	-		7.05 ± 5.87		10.88 ± 8.53		**< 0.001**
Heart Failure incidence, n (%)	2,929 (2.96)		479 (5.19)		831 (3.49)		**< 0.001**
Years post consent (years)	8.03 ± 3.30		7.64 ± 3.46		8.07 ± 3.47		**0.030**

HDL-C: High-density lipoprotein cholesterol; LDL-C: Low-density lipoprotein cholesterol; IPAQ: International Physical Activity Questionnaire; ARIC-HF: Atherosclerosis Risk in Communities – Heart Failure; PCP-HF: Pooled Cohort Equations to Prevent Heart Failure; HFA-ICOS: Cardio-Oncology Risk Assessment.

*p-value indicates differences between participants with breast cancer or lymphoma and other cancer types.

### HF incidence

**Fig. 1:**
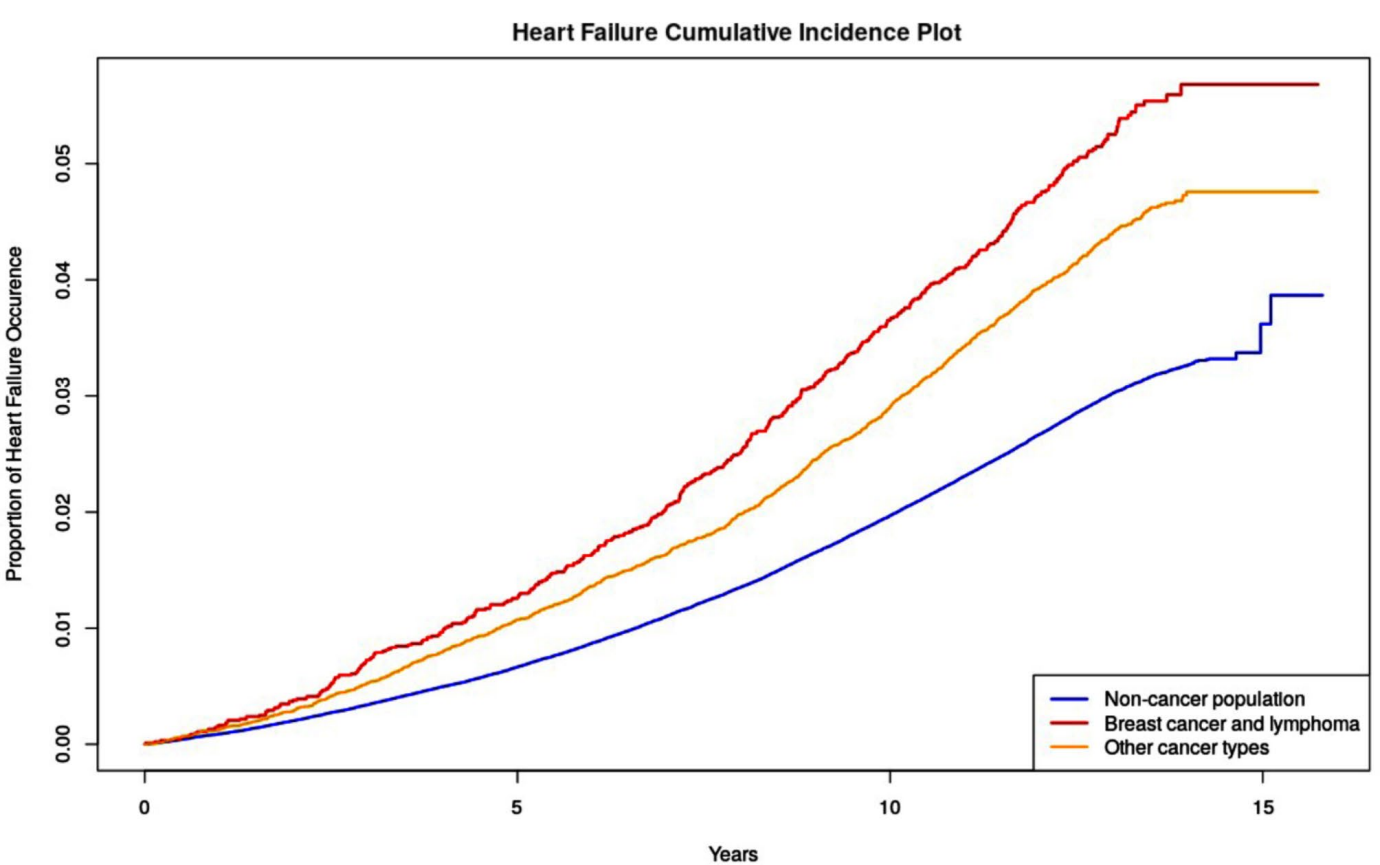
The cumulative incidence plot of heart failure among UK Biobank participants

The overall HF incidence was 4.51% among cancer survivors at an average of 7.76 years follow-up. HF was identified in 5.19% of the breast cancer and lymphoma survivors, significantly more than 3.49% within other cancer survivors and 2.96% within the controls (Fig. 1).

**Fig. 2:**
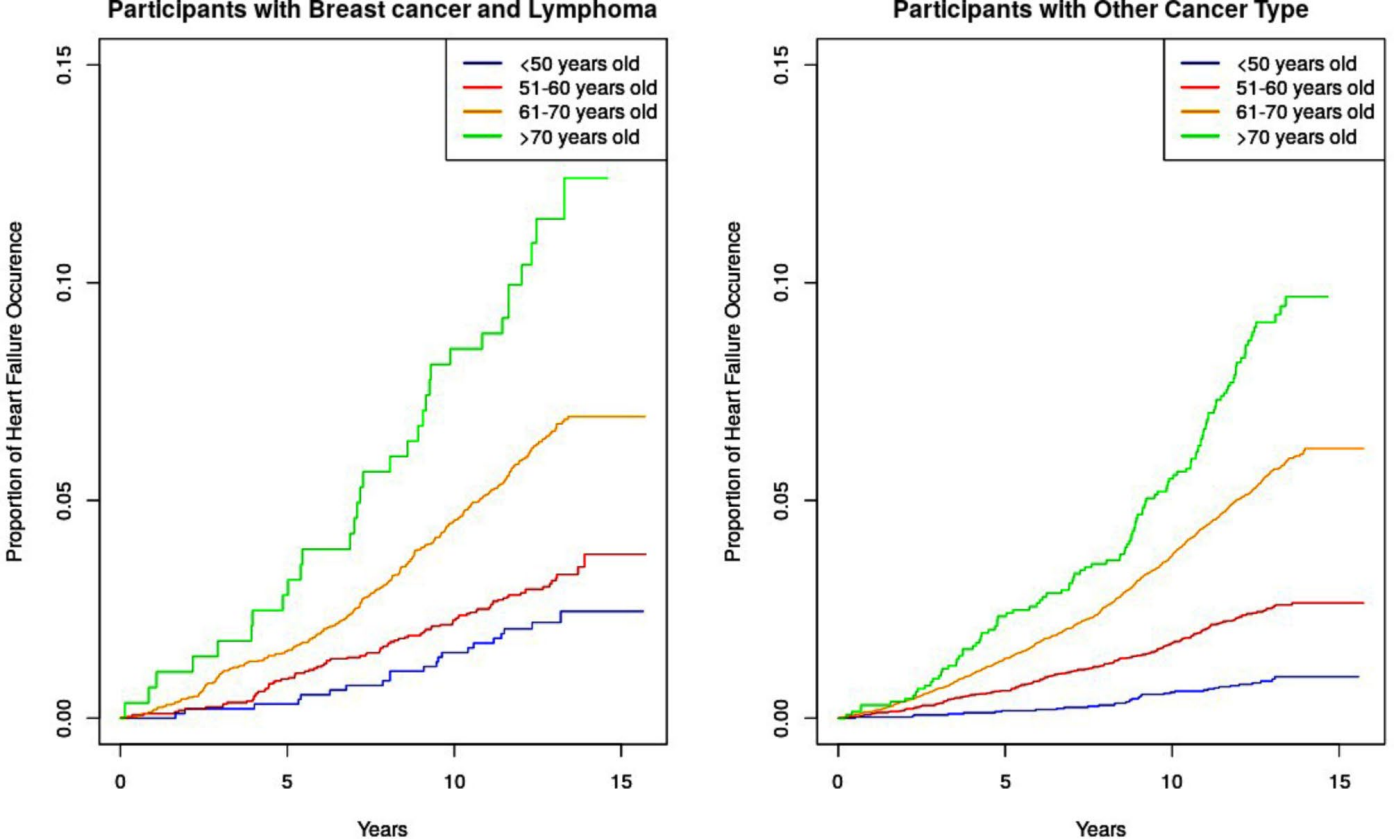
The cumulative incidence plot of heart failure among cancer survivors, stratified according to age range

Figure 2 demonstrates the cumulative incidence of HF, stratified by age range, among [i] breast cancer and lymphoma participants and [ii] participants with other cancer types. It is noticeable that among participants aged above 70 years, incident HF is significantly higher (*p* < 0.001) among breast cancer and lymphoma participants (11.3%) than participants with other cancer types (8.97%). Stratifying the cancer survivors based on the duration of cancer diagnoses prior consent, the incident HF in each subgroup is reported in Supplementary Table 6. Overall, there is no statistically significant differences in the incident HF across different duration of cancer diagnoses prior consent.

### Prediction of incident HF

**Fig. 3:**
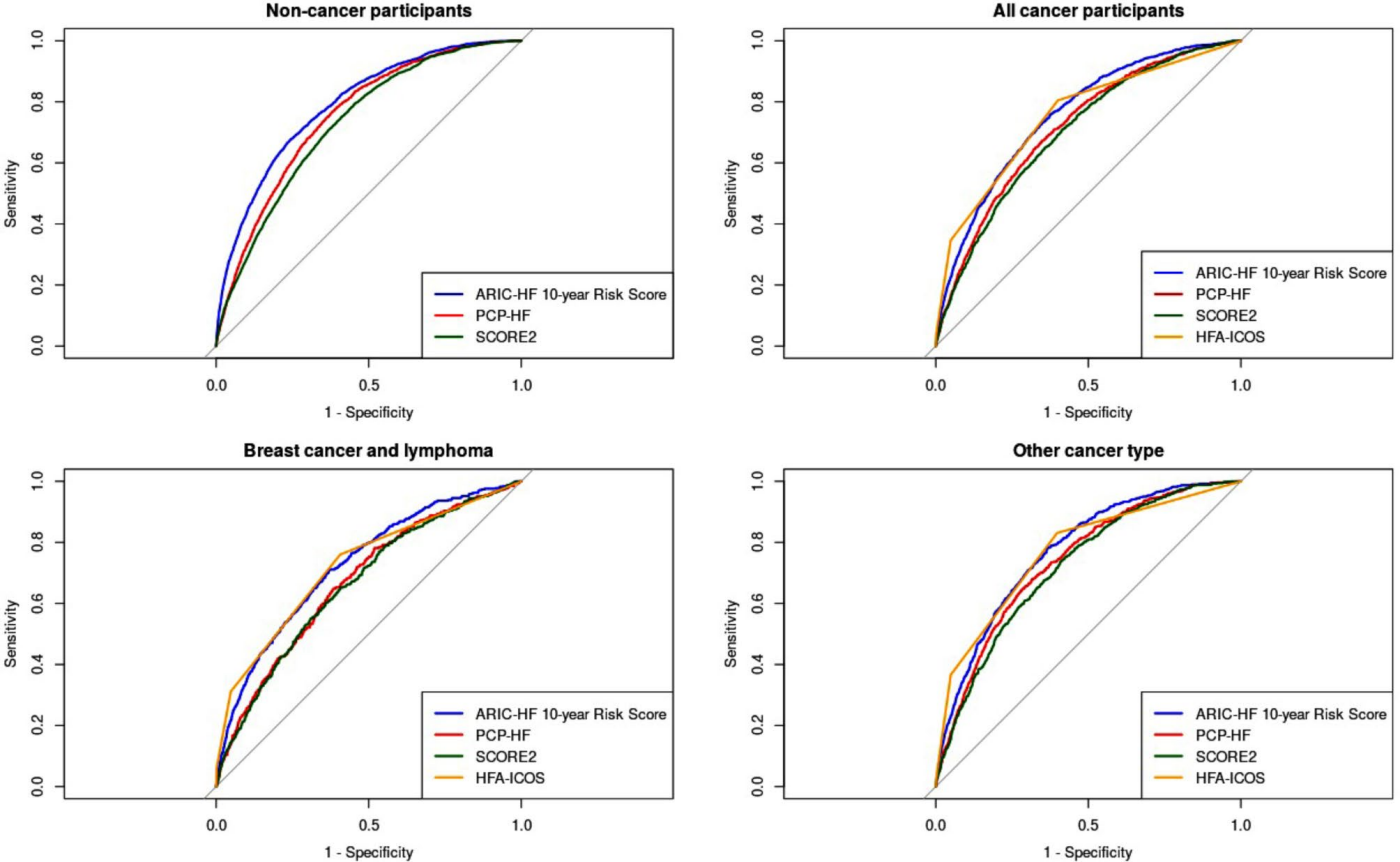
The receiver operating characteristics curve for each CVD risk assessment tool in predicting heart failure incidence. CVD: Cardiovascular disease; ARIC-HF: Atherosclerosis Risk in Communities – Heart Failure; PCP-HF: Pooled Cohort Equations to Prevent Heart Failure; HFA-ICOS: Cardio-Oncology Risk Assessment.

The performances of the three risk assessment tools are compared in Table 2. Overall, HFA-ICOS (AUC: 0.753) and ARIC-HF (AUC: 0.757, *p* = 0.548) outperformed PCP-HF (AUC: 0.717, *p* < 0.001) in predicting incident HF among cancer survivors. In addition, it is noticeable that all three CVD risk assessment tools performed worse in the subpopulation of breast cancer and lymphoma participants. The ROC curves of the CVD risk assessment tools are illustrated in Fig. 3.

**Table 2:** The area under the curve for each CVD risk assessment tool in predicting heart failure

	Non-cancer patients		All cancer participants		Breast cancer and lymphoma		Other cancer types
HFA-ICOS	-		0.753 (0.739–0.766)		0.723 (0.700-0.747)		0.769 (0.753–0.785)
ARIC-HF	0.792 (0.784-0.800)		0.757 (0.744–0.770)		0.725 (0.701–0.749)		0.773 (0.758–0.789)
PCP-HF	0.754 (0.745–0.763)		0.717 (0.702–0.732)		0.671 (0.643–0.698)		0.741 (0.723–0.758)

CVD: Cardiovascular disease; HFA-ICOS: Cardio-Oncology Risk Assessment; ARIC-HF: Atherosclerosis Risk in Communities – Heart Failure; PCP-HF: Pooled Cohort Equations to Prevent Heart Failure.

In comparison, the ARIC-HF and PCP-HF performed significantly better in HF prediction among non-cancer participants (AUC: 0.792 and 0.754, *p* < 0.001, respectively) than cancer participants. HFA-ICOS was not calculated among non-cancer participants given it being a cancer-specific HF risk assessment.

Based on Youden’s index to obtain the optimal balance of sensitivity and specificity, the HFA-ICOS was shown to have the highest sensitivity (80.48%) at its ideal cut-off point compared to the remaining CVD risk assessment tools among all cancer participants (ARIC-HF: 75.85%, *p* < 0.001; PCP-HF: 66.89%, *p* < 0.001). Similar output was identified in the subpopulations of breast cancer and lymphoma participants and those with other cancer types (Table 3). By contrast, PCP-HF showed the highest sensitivity (76.74%) at its ideal cut-off point among non-cancer participants.

**Table 3:** Sensitivity and specificity of each CVD risk assessment tool in predicting heart failure based on Youden’s index

	Non-cancer participants				All cancer participants			Breast cancer and lymphoma			Other cancer types	
	Sensitivity	Specificity		Sensitivity		Specificity		Sensitivity	Specificity		Sensitivity	Specificity
HFA-ICOS	-	-		80.48%		59.99%		76.00%	59.36%		83.09%	60.23%
ARIC-HF	67.67%	75.64%		75.85%		62.45%		71.11%	62.84%		78.25%	63.34%
PCP-HF	76.74%	61.91%		66.89%		65.48%		64.84%	61.60%		67.92%	68.49%

CVD: Cardiovascular disease; HFA-ICOS: Cardio-Oncology Risk Assessment; ARIC-HF: Atherosclerosis Risk in Communities – Heart Failure; PCP-HF: Pooled Cohort Equations to Prevent Heart Failure

As a screening process needs to provide high sensitivity, if necessary at the cost of specificity, we repeated the above analysis with sensitivity fixed at 80% and 90% (Table 4). Among all cancer participants, HFA-ICOS showed the highest specificity (60.36%) among the three CVD risk assessment tools at a sensitivity of 80%. In contrast, a default sensitivity of 90% showed that ARIC-HF is the best in excluding those who do not develop incident HF (41.33%) whilst HFA-ICOS demonstrated the worst performance (30.73%). Table 4 also demonstrates that the CVD risk assessment tool with the highest specificity at a default sensitivity value of 80% and 90% was ARIC-HF (49.57% and 33.43% respectively) among participants with breast cancer and lymphoma.

**Table 4:** Specificity of each CVD risk assessment tool in predicting heart failure at a level producing a sensitivity of 80% and 90%

	Non-cancer participants			All cancer participants			Breast cancer and lymphoma			Other cancer types	
	Sens = 80%	Sens = 90%		Sens = 80%	Sens = 90%		Sens = 80%	Sens = 90%		Sens = 80%	Sens = 90%
HFA-ICOS	-	-		60.36%	30.73%		49.47%	24.73%		62.55%	35.61%
ARIC-HF	61.02%	45.33%		56.18%	41.33%		49.57%	33.43%		59.66%	45.20%
PCP-HF	58.12%	41.92%		50.59%	34.19%		43.22%	25.44%		54.09%	37.55%

CVD: Cardiovascular disease; HFA-ICOS: Cardio-Oncology Risk Assessment; ARIC-HF: Atherosclerosis Risk in Communities – Heart Failure; PCP-HF: Pooled Cohort Equations to Prevent Heart Failure

Table 5 shows the association between each clinical characteristics and HF incidence in a competing risk regression model. Among breast cancer and lymphoma participants, the ARIC-HF and PCP-HF showed an SHR of 1.21 (95%CI: 1.17–1.24) and 1.25 (95%CI: 1.21–1.29), respectively. On the other hand, both of these CVD risk assessment tools had an SHR of 1.12 (95%CI: 1.11–1.14) and 1.21 (95%CI: 1.18–1.24), respectively among participants with other cancer types.

**Table 5:** Univariable competing risk regression analysis on heart failure incidence

	All cancer participants	Breast cancer and lymphoma	Other cancer type
	SHR (95%CI)	SHR (95%CI)	SHR (95%CI)
Age, per 1-year increase	**1.08 (1.07–1.09)**	**1.07 (1.06–1.09)**	**1.10 (1.09–1.11)**
Male	**1.69 (1.50–1.90)**	**3.10 (2.54–3.78)**	**2.01 (1.82–2.23)**
Alcohol consumption			
Never	Ref	Ref	Ref
Previous	1.25 (0.87–1.79)	1.24 (0.74–2.09)	1.31 (0.96–1.77)
Current	0.85 (0.65–1.12)	0.81 (0.55–1.19)	0.78 (0.62–0.99)
Smoking status			
Never	Ref	Ref	Ref
Previous	** 1.39 (1.23–1.58)**	** 1.45 (1.20–1.75)**	** 1.65 (1.48–1.85)**
Current	** 1.56 (1.29–1.87)**	** 1.37 (1.00-1.88)**	** 2.02 (1.73–2.35)**
Body mass index, per 1 kg/m2 increase	**1.07 (1.06–1.09)**	**1.08 (1.06–1.10)**	**1.07 (1.07–1.08)**
Total cholesterol, per 1mmol/L increase	**0.77 (0.72–0.81)**	**0.80 (0.72–0.87)**	**0.71 (0.67–0.74)**
HDL-C, per 1mmol/L increase	** 0.52 (0.43–0.63)**	** 0.49 (0.36–0.66)**	** 0.41 (0.34–0.49)**
LDL-C, per 1mmol/L increase	** 0.73 (0.68–0.79)**	** 0.78 (0.69–0.88)**	** 0.66 (0.62–0.71)**
Systolic blood pressure, per 1mmHg increase	**1.01 (1.01–1.02)**	**1.01 (1.01–1.02)**	**1.01 (1.01–1.01)**
Diastolic blood pressure, per 1mmHg increase	**1.01 (1.00-1.01)**	1.01 (1.00-1.01)	1.00 (0.99-1.00)
Heart rate, per 1 bpm increase	**1.01 (1.00-1.01)**	1.01 (1.00-1.02)	**1.01 (1.01–1.02)**
Diabetes	**2.45 (2.05–2.93)**	**2.45 (1.84–3.26)**	**3.27 (2.84–3.76)**
Coronary heart disease	**6.18 (5.39–7.09)**	**6.70 (5.28–8.49)**	**5.96 (5.29–6.71)**
Hypertension	**4.00 (2.76–5.82)**	**3.30 (1.72–6.33)**	**2.98 (2.05–4.33)**
Chronic kidney disease	**4.11 (2.73–6.18)**	**10.8 (7.26-16.0)**	**6.08 (4.42–8.37)**
Obesity	**3.31 (1.41–7.76)**	-	**2.34 (1.00-5.44)**
Medications			
Insulin	** 3.47 (2.54–4.74)**	** 3.59 (2.13–6.06)**	** 4.49 (3.52–5.73)**
Blood pressure medication	** 2.74 (2.44–3.08)**	** 2.59 (2.16–3.10)**	** 3.07 (2.78–3.40)**
Cholesterol lowering medication	** 2.95 (2.62–3.32)**	** 2.45 (2.02–2.98)**	** 3.15 (2.84–3.49)**
ARIC-HF	**1.12 (1.10–1.13)**	**1.21 (1.17–1.24)**	**1.12 (1.11–1.14)**
PCP-HF	**1.20 (1.17–1.22)**	**1.25 (1.21–1.29)**	**1.21 (1.18–1.24)**
HFA-ICOS			
Low	Ref	Ref	Ref
Medium	** 3.89 (3.27–4.64)**	** 3.03 (2.42–3.81)**	** 4.34 (3.72–5.08)**
High	** 19.0 (15.9–22.7)**	** 11.8 (9.11–15.2)**	** 20.9 (17.8–24.4)**
Very high	** 84.4 (61.7–115)**	** 66.8 (42.9–104)**	** 57.8 (41.3–80.9)**

SHR: Subdistribution hazard ratio; CI: Confidence interval; HDL-C: High-density lipoprotein cholesterol; LDL-C: Low-density lipoprotein cholesterol; ARIC-HF: Atherosclerosis Risk in Communities – Heart Failure; PCP-HF: Pooled Cohort Equations to Prevent Heart Failure; HFA-ICOS: Cardio-Oncology Risk Assessment

Death was set as a competing risk. Bold SHR indicates *p*-value < 0.05

**Table 6:** Multivariable competing risk regression analysis on heart failure incidence

	All cancer participants	Breast cancer and lymphoma	Other cancer type
	SHR (95%CI)	SHR (95%CI)	SHR (95%CI)
Age, per 1-year increase	**1.02 (1.01–1.03)**	**1.03 (1.01–1.05)**	**1.04 (1.02–1.05)**
Male	-	**2.41 (1.93–2.99)**	**1.21 (1.08–1.35)**
Body mass index, per 1 kg/m2 increase	**1.03 (1.02–1.05)**	**1.05 (1.03–1.07)**	**1.03 (1.02–1.05)**
Heart rate, per 1 bpm increase	**1.01 (1.00-1.01)**	**1.01 (1.00-1.02)**	**1.01 (1.01–1.02)**
Coronary heart disease	**2.03 (1.68–2.44)**	**2.43 (1.78–3.31)**	**1.95 (1.66–2.28)**
Hypertension	**1.78 (1.22–2.60)**	**2.23 (1.23–4.01)**	-
Chronic kidney disease	-	**5.55 (3.52–8.75)**	**2.32 (1.58–3.40)**
Medications			
Insulin	-	-	** 1.50 (1.13-2.00)**
Blood pressure medication	** 1.28 (1.12–1.47)**	** 1.42 (1.15–1.74)**	** 1.37 (1.22–1.55)**
HFA-ICOS			
Low	Ref	Ref	Ref
Medium	** 2.80 (2.26–3.48)**	** 1.72 (1.30–2.27)**	** 2.55 (2.09–3.10)**
High	** 10.2 (7.96-13.0)**	** 4.45 (3.17–6.24)**	** 8.74 (6.99–10.9)**
Very high	** 62.5 (43.8–89.3)**	** 57.6 (35.3–93.9)**	** 32.5 (21.7–48.6)**

SHR: Subdistribution hazard ratio; CI: Confidence interval; HFA-ICOS: Cardio-Oncology Risk Assessment

Death was set as a competing risk. Bold SHR indicates *p*-value < 0.05

After adjusting for other confounding variables, none of the three CVD risk assessment tools were significantly associated with HF incidence (Table 6). HFA-ICOS was shown to be the only significant predictor of the three CVD risk assessment tools in the competing risk regression model in both subgroups of participants with breast cancer and lymphoma or other cancer types.

## Discussion

The results of this study show that incident HF is greater among participants with breast cancer and lymphoma than those with other cancer types. In addition, our study shows that HFA-ICOS and ARIC-HF had a greater predictive performance than PCP-HF in predicting incident HF among all cancer participants. Specifically, HFA-ICOS demonstrated the highest specificity while ARIC-HF showed the highest sensitivity at their respective ideal cut- points. Nevertheless, none of the three CVD risk assessment tools was significantly superior in their discriminating performance in predicting incident HF among cancer participants. Additionally, their predictive performances were reduced among breast cancer and lymphoma participants.

### Prediction of HF in survivors

The development of CVD is greatest among cancer survivors who have received potentially cardio-toxic cancer treatments [10]. In this respect, the HF risk associated with use of anthracycline chemotherapy [11] is being augmented by potentially cardiotoxic molecular therapies. The emergence of cardio-oncology services over the past decade [12, 13] has particularly focused on the risk of LV dysfunction and HF occurring at the time of chemotherapy. A Dutch study showed that cancer treatments, including mediastinal radiotherapy and anthracycline-containing chemotherapy, as well as the corresponding dosage were associated with HF and other CVDs [14]. However, as potentially toxic therapies also sensitize the myocardium to subsequent injuries, there may be benefit in extending HF surveillance into survivorship.

Our study isolated participants with breast cancer and lymphoma from other cancer types due to the known risk of HF during survivorship, perhaps reflecting the ongoing use of anthracyclines [15], which display dose-dependent cardiotoxic effects and congestive heart failure [16–18]. A meta-analysis conducted in 2013 showed that after a median follow-up duration of 9 years, 6% of patients treated with anthracyclines demonstrated clinical cardiotoxic effects, and 18% demonstrated subclinical cardiotoxic effects [19]. Given the increased CVD risk from anthracycline-based chemotherapy, breast cancer and lymphoma patients who also underwent radiotherapy would pose an even greater risk for incident HF, and therefore additional attention is required to monitor their cardiovascular function [20]. 

### HF risk scores

HFA-ICOS was developed by the Cardio-Oncology Study Group of the Heart Failure Association of the European Society of Cardiology and the International Cardio-Oncology Society. Its aim was to provide baseline cardiovascular risk stratification to stratify groups of cancer patients before initiation of potentially cardiotoxic cancer treatment [5]. A previous study conducted among HER2-positive breast cancer patients reported an AUC of 0.64 in the prediction of overall treatment-related cardiotoxicity using HFA-ICOS [21], which is slightly lower than what we reported in this study. Cancer patients were stratified into low, medium, high and very high risk based on the accumulation of risk factors with a predetermined risk level. In addition, HFA-ICOS is the only one among the three evaluated tools that is designed specifically for cancer patients and incorporated cancer treatment into its evaluation. It is easy to implement for both research and clinical setting and our study showed that it has the highest predictive value among the other CVD risk assessment tools. However, the simplicity of this CVD risk assessment tool comes with a key limitation. The weighting of each CVD risk factor is predetermined and dichotomised into presence/absence, reducing the information provided to the overall risk stratification. On the other hand, ARIC-HF treats factors such as age, blood pressure and biomarkers level as a continuous variable with increasing risk [6]. Therefore, while the HFA-ICOS is intuitive to implement, further research is required to look into the overall performance in clinical settings and its feasibility.

While HFA-ICOS showed the highest predictive performance, our study also showed that ARIC-HF is a clinically valid prognostic tool for assessing HF risk in survivors. ARIC-HF combines key risk factors for incident HF, including age, sex, race, comorbidities, medications and NT-proBNP [6]. The suboptimal performance of ARIC-HF in predicting incident HF among cancer patients could potentially be attributed to the lack of cancer treatment in the calculation. Given that cancer treatments such as chemotherapy and radiotherapy pose additional CVD risk to cancer patients, the inclusion of such variables into the risk assessment tool may increase its overall prognostic value. As breast cancer and lymphoma patients are the most likely to have exposure to potentially cardiotoxic agents, this may also explain the lower predictive performance of HF risk scores in these patients.

ARIC-HF demonstrated the highest sensitivity at its ideal cut-off value, which may be more useful and cost-effective to be implemented in clinical settings. On the other hand, while HFA-ICOS showed the highest predictive performance, it was also shown to be better in ruling out those who are at lower risk (highest specificity) than identifying those who are at higher risk (low sensitivity). This might not provide much clinical value in the context of identifying cancer patients who are at greater risk of incident HF to provide regular checkup and initiate cardioprotective treatment if necessary [22]. However, we acknowledge that our assessment of participants’ HF risk is based on cross-sectional analysis, and the lack of data on cancer treatment in our database could significantly impact these findings. The 2022 European Society of Cardiology (ESC) guidelines on cardio-oncology emphasize that cancer treatment can substantially alter a survivor’s risk profile. Therefore, a dynamic evaluation is crucial to accurately determine survivors’ ongoing risk and guide subsequent cardiovascular disease (CVD) management plans [23]. 

Our study also showed that PCP-HF was inferior in the prediction of incident HF among cancer patients. The first validation of the risk assessment tool reported in its original study shown an AUC of 0.74 and 0.73 in white men and women respectively [7], which is relatively lower compared to an AUC of 0.797 reported by the ARIC-HF in its first external validation work. Moreover, it has yet to be validated widely across different cohorts, not to mention in cancer population. Hence, it is within expectation that they may not perform as well in the prediction of incident HF among cancer patients in our study.

With death as a competing risk, the univariable regression analyses revealed that traditional CVD risk factors—such as age, sex, smoking status, blood pressure, and comorbidities—are significantly associated with HF incidence. This finding is unsurprising, given that these factors have been extensively studied and are commonly incorporated into various risk scores to accurately reflect patients’ HF risk [24]. Interestingly, in the univariable analysis, an increase in total cholesterol levels appeared to be protective against HF incidence. This effect may be influenced by the protective role of elevated HDL cholesterol levels (unadjusted SHR: 0.77, [95% CI: 0.72–0.81]), though we lack information on non-HDL cholesterol levels. Additionally, hyperlipidemia is often associated with statin use, which is known to be protective of HF incidence. Alternatively, this could represent a false positive association due to multiple comparisons. Nonetheless, this positive association diminished after adjusting for other risk factors in the multivariable regression analysis. Notably, the multivariable analysis also revealed that, independent of all conventional CVD risk factors, cancer survivors classified as *medium*, *high*, or *very high* risk based on HFA-ICOS were at significantly higher risk of HF occurrence post-assessment, underscoring the strong discriminative ability of HFA-ICOS.

### Study limitations

While the UK Biobank affords the opportunity of studying outcomes in large numbers of patients, it has some disadvantages. Participants in the UK Biobank are mostly Caucasians, and this could potentially limit the generalisability of our findings in other ethnicities and regions. Secondly, our study design in excluding participants with previous HF diagnosis eliminates one of the key determinants for risk classification in HFA-ICOS, which could potentially result in an under-estimation of its overall predictive accuracy. In addition, key clinical data including biomarkers and cancer treatments are not fully available, limiting the overall accuracy in survivors’ risk classification. Furthermore, the utilisation of ICD-10 codes in confirming diagnoses would potentially result in diseases, especially chronic diseases, being underdiagnosed. Lastly, cancer patients who consented for the UK-Biobank study might generally be healthier than those who are frailer and of greater risk of any CVDs, hence limiting its external validity.

## Conclusions

Participants with breast cancer and lymphoma are of increased risk of incident HF. HFA-ICOS and ARIC-HF scores outperform PCP-HF in predicting incident HF among cancer participants, specifically ruling out those who are lower risk of incident HF. However, none of them demonstrates discriminative ability that is significantly superior in the prognosis of incident HF among participants with cancer, whether that is breast cancer and lymphoma or other cancer types. Our study shows that a cancer-specific HF risk prediction tool, especially breast cancer and lymphoma patients, is warranted in order to provide cancer patients with timely and cost-effective care.

## Electronic supplementary material

Below is the link to the electronic supplementary material.

Supplementary Material 1

## Data Availability

The data that support the findings of this study are available from the UK-Biobank study team but restrictions apply to the availability of these data, which were used under license for the current study, and so are not publicly available. Data are however available from the authors upon reasonable request and with permission of the UK-biobank study team.

## Abbreviations

ARIC: Atherosclerosis Risk in Communities
AUC: Area under the curve
CI: Confidence interval
CVD: Cardiovascular disease
HF: Heart failure
HFA-ICOS: Heart Failure Association-International Cardio-Oncology Society
ICD: International Classification of Diseases
IPAQ: International Physical Activity Questionnaire
PCP-HF: Pooled Cohort Equations to Prevent Heart Failure
ROC: Receiver operating characteristics
SHR: Subdistribution hazard ratio
