# Supplementary material for: Comparison of heart failure risk assessment tools among cancer survivors
Source: Cardiooncology. 2024 Oct 11;10:67. doi: 10.1186/s40959-024-00267-5 (PMC11468191; doi:10.1186/s40959-024-00267-5)
Supplement: Supplementary file 1 — Supplementary Material 1 [file 40959_2024_267_MOESM1_ESM.docx]

STROBE Statement—Checklist of items that should be included in reports of ***cohort studies***

|  | Item No | Recommendation | | Page No |
| --- | --- | --- | --- | --- |
| **Title and abstract** | 1 | (*a*) Indicate the study’s design with a commonly used term in the title or the abstract | | NA |
|  |  | (*b*) Provide in the abstract an informative and balanced summary of what was done and what was found | | 2 |
| Introduction | | | | |
| Background/rationale | 2 | Explain the scientific background and rationale for the investigation being reported | | 3 |
| Objectives | 3 | State specific objectives, including any prespecified hypotheses | | 3 |
| Methods | | | | |
| Study design | 4 | Present key elements of study design early in the paper | | 3 |
| Setting | 5 | Describe the setting, locations, and relevant dates, including periods of recruitment, exposure, follow-up, and data collection | | 3 |
| Participants | 6 | (*a*) Give the eligibility criteria, and the sources and methods of selection of participants. Describe methods of follow-up | | 3 |
|  |  | (*b*) For matched studies, give matching criteria and number of exposed and unexposed | | NA |
| Variables | 7 | Clearly define all outcomes, exposures, predictors, potential confounders, and effect modifiers. Give diagnostic criteria, if applicable | | 3-4 |
| Data sources/ measurement | 8* | For each variable of interest, give sources of data and details of methods of assessment (measurement). Describe comparability of assessment methods if there is more than one group | | 3-4 |
| Bias | 9 | Describe any efforts to address potential sources of bias | | NA |
| Study size | 10 | Explain how the study size was arrived at | | 5 |
| Quantitative variables | 11 | Explain how quantitative variables were handled in the analyses. If applicable, describe which groupings were chosen and why | | 3-4 |
| Statistical methods | 12 | (*a*) Describe all statistical methods, including those used to control for confounding | | 4 |
|  |  | (*b*) Describe any methods used to examine subgroups and interactions | | 4-5 |
|  |  | (*c*) Explain how missing data were addressed | | 5 |
|  |  | (*d*) If applicable, explain how loss to follow-up was addressed | | NA |
|  |  | (*e*) Describe any sensitivity analyses | | NA |
| Results | | | |  |
| Participants | 13* | (a) Report numbers of individuals at each stage of study—eg numbers potentially eligible, examined for eligibility, confirmed eligible, included in the study, completing follow-up, and analysed | | 5 |
|  |  | (b) Give reasons for non-participation at each stage | | 5 |
|  |  | (c) Consider use of a flow diagram | | NA |
| Descriptive data | 14* | (a) Give characteristics of study participants (eg demographic, clinical, social) and information on exposures and potential confounders | | 5, 12-14 |
|  |  | (b) Indicate number of participants with missing data for each variable of interest | | 12-14 |
|  |  | (c) Summarise follow-up time (eg, average and total amount) | | 5, 14 |
| Outcome data | 15* | Report numbers of outcome events or summary measures over time | | 5, 14 |
| Main results | 16 | (*a*) Give unadjusted estimates and, if applicable, confounder-adjusted estimates and their precision (eg, 95% confidence interval). Make clear which confounders were adjusted for and why they were included | 5-6 | |
|  |  | (*b*) Report category boundaries when continuous variables were categorized | NA | |
|  |  | (*c*) If relevant, consider translating estimates of relative risk into absolute risk for a meaningful time period | NA | |
| Other analyses | 17 | Report other analyses done—eg analyses of subgroups and interactions, and sensitivity analyses | 6 | |
| Discussion | | | | |
| Key results | 18 | Summarise key results with reference to study objectives | 6 | |
| Limitations | 19 | Discuss limitations of the study, taking into account sources of potential bias or imprecision. Discuss both direction and magnitude of any potential bias | 9 | |
| Interpretation | 20 | Give a cautious overall interpretation of results considering objectives, limitations, multiplicity of analyses, results from similar studies, and other relevant evidence | 6-8 | |
| Generalisability | 21 | Discuss the generalisability (external validity) of the study results | 9 | |
| Other information | | | | |
| Funding | 22 | Give the source of funding and the role of the funders for the present study and, if applicable, for the original study on which the present article is based | 10 | |

Supplementary Table 1: Baseline Cardio-Oncology Risk Assessment tool.

| Risk factor | Score | Level of Evidence |
| --- | --- | --- |
| ***Previous cardiovascular disease*** | | |
| Heart failure or cardiomyopathy | Very High | C |
| Myocardial infarction or CABG | High | B |
| Stable angina | High | B |
| Severe valvular heart disease | High | C |
| Baseline LVEF <50% | High | C |
| Borderline LVEF 50-54% | Medium^2^ | B |
| Arrhythmia | Medium^2^ | C |
| ***Cardiac biomarkers*** | | |
| Elevated baseline troponin | Medium^2^ | B |
| Elevated baseline BNP or NT-proBNP | Medium^2^ | C |
| ***Demographic and cardiovascular risk factors*** | | |
| Age ≥ 80 years | High | B |
| Age 65-79 years | Medium^2^ | B |
| Hypertension | Medium^1^ | B |
| Diabetes mellitus | Medium^1^ | C |
| Chronic kidney disease | Medium^1^ | C |
| ***Current cancer treatment regimen*** | | |
| Includes Anthracycline before HER2-targeted therapy | Medium^1^ | B |
| ***Previous cardiotoxic cancer treatment*** | | |
| Prior trastuzumab cardiotoxicity | Very High | C |
| Prior (remote) anthracycline exposure*** | Medium^2^ | B |
| Prior radiotherapy to left chest or mediastinum | Medium^2^ | C |
| ***Lifestyle risk factors*** | | |
| Current smoker or significant smoking history | Medium^1^ | C |
| Obesity (BMI>30) | Medium^1^ | C |

BMI: Body mass index; BNP: brain natriuretic peptide; CABG: coronary artery bypass graft; LVEF: left ventricular ejection fraction; NT-proBNP: N-terminal pro-brain natriuretic peptide.

Low risk: no risk factor OR one MEDIUM^1^ risk factor.

Medium risk: MEDIUM risk factors with a total of 2-4 points.

High risk: MEDIUM risk factors with a total of ≥5 points OR any HIGH risk factor.

Very high risk: any VERY HIGH risk factor.

Supplementary Table 2: Regression coefficients from multivariable models fit with the variables in the ARIC-HF risk score.

|  | White Male | White Female | Black Male | Black Female |
| --- | --- | --- | --- | --- |
| Age (year) (Field ID 33) | 0.072 | 0.081 | 0.013 | 0.069 |
| Heart rate (bpm) (Field ID 102) | 0.023 | 0.031 | 0.031 | 0.021 |
| Systolic BP (mmHg) (Field ID 4080) | 0.001 | 0.006 | 0.005 | -0.003 |
| BP lowering medication (Field ID 6177 & 6153) | 0.194 | 0.27 | 0.326 | 0.081 |
| Diabetes (Field ID 2443 & 41202) | 0.62 | 0.795 | 0.83 | 1.042 |
| Coronary Heart Disease (Field ID 41202) | 0.731 | 0.531 | 0.682 | 0.36 |
| Current smoker (Field ID 20116) | 0.971 | 1.055 | 0.882 | 0.241 |
| Former smoker (Field ID 20116) | 0.327 | 0.444 | 0.471 | 0.16 |
| Body mass index (kg/m^2^) (Field ID 21001) | 0.073 | 0.062 | 0.032 | 0.043 |
| Baseline Survival (S_0_) | 0.99334 | 0.99671 | 0.988 | 0.99095 |

ARIC-HF: Atherosclerosis Risk in Communities – Heart Failure; BP: Blood pressure.

Supplementary Table 3: Race- and sex-specific equation parameters for estimation of 10-year risk of heart failure derived from the Lifetime Risk Pooling Project for the Modified Pooled Cohort Equations to Prevent Heart Failure (PCP-HF) without QRS duration.

|  | White Male | White Female | Black Male | Black Female |
| --- | --- | --- | --- | --- |
| Ln Age (year) | 42.5 | 18.15 | 2.99 | 50.32 |
| Ln Age, Squared | -0.97 | N/A | N/A | N/A |
| Ln Treated Systolic BP (mmHg) | 0.97 | 10.85 | 2.4 | 29 |
| Ln Age x Ln Treated Systolic BP | N/A | -2.47 | N/A | -6.57 |
| Ln Untreated Systolic BP (mmHg) | 0.85 | 9.71 | 2.26 | 28.09 |
| Ln Age x Ln Untreated Systolic BP | N/A | -2.21 | N/A | -6.37 |
| Current Smoker | 0.73 | 10.95 | 1.49 | 0.75 |
| Ln Age x Current Smoker | N/A | -2.5 | -0.21 | N/A |
| Ln Treated glucose (mg/dL) | 0.92 | 1.03 | 0.63 | 0.99 |
| Ln Untreated glucose (mg/dL) | 0.8 | 0.9 | 0.55 | 0.83 |
| Ln Total Cholesterol (mg/dL) | 0.46 | N/A | N/A | 0.26 |
| Ln HDL-C (mg/dL) | -0.42 | -0.07 | -0.8 | N/A |
| Ln BMI (kg/m^2^) | 37 | 1.37 | 1.21 | 19.75 |
| Ln Age x Ln BMI | -8.77 | N/A | N/A | -4.6 |
| Mean Coefficient x Value (MeanCV) | 169.25 | 85.03 | 26.3 | 223.05 |
| Baseline Survival (S_0_) | 0.98743 | 0.99331 | 0.98297 | 0.99241 |

BMI: Body mass index; BP: blood pressure; HDL: high density lipoprotein.

Supplementary Table 4: Contingency table between blood pressure lowering medication, ICD10 coded hypertension diagnoses and elevated blood pressure.

|  |  | Blood pressure lowering medication | |
| --- | --- | --- | --- |
|  |  | No | Yes |
| Hypertension (IC10 coded) | No | 103,483 | 27,760 |
|  | Yes | 391 | 494 |

|  |  | Blood pressure lowering medication | |
| --- | --- | --- | --- |
|  |  | No | Yes |
| Elevated blood pressure | No | 59,203 | 9,961 |
|  | Yes | 38,965 | 16,636 |

Supplementary Table 5: Characteristics of participants prior to propensity matching.

|  | Non-cancer participants  (n = 440,813) |  | Breast cancer and lymphoma  (n = 9,232) |  | Other cancer types  (n = 34,488) |  | *p* |
| --- | --- | --- | --- | --- | --- | --- | --- |
| Age (y) | 56.72 ± 8.11 |  | 59.86 ± 6.98 |  | 60.07 ± 7.42 |  | 0.012 |
| Female, n (%) | 234,967 (53.30) |  | 8,103 (87.77) |  | 20,413 (59.19) |  | <0.001 |
| Ethnicity, n (%) |  |  |  |  |  |  | 0.003 |
| White | 414,121 (93.94) |  | 8,930 (96.73) |  | 33,567 (97.33) |  |  |
| Asian | 9,062 (2.06) |  | 80 (0.87) |  | 202 (0.59) |  |  |
| Black | 7,264 (1.65) |  | 77 (0.83) |  | 270 (0.78) |  |  |
| Chinese | 1,437 (0.33) |  | 15 (0.16) |  | 47 (0.14) |  |  |
| Mixed | 2,654 (0.60) |  | 48 (0.52) |  | 124 (0.36) |  |  |
| Other | 4,141 (0.94) |  | 54 (0.58) |  | 146 (0.42) |  |  |
| Alcohol consumption, n (%) |  |  |  |  |  |  | <0.001 |
| Never | 19,493 (4.42) |  | 461 (4.99) |  | 1,379 (4.00) |  |  |
| Previous | 15,476 (3.51) |  | 385 (4.17) |  | 1,385 (4.02) |  |  |
| Current | 404,698 (91.81) |  | 8,364 (90.60) |  | 31,669 (91.83) |  |  |
| Smoking status, n (%) |  |  |  |  |  |  | <0.001 |
| Never | 242,286 (54.96) |  | 5,012 (54.29) |  | 17,083 (49.53) |  |  |
| Previous | 149,841 (33.99) |  | 3,391 (36.73) |  | 13,595 (39.42) |  |  |
| Current | 404,698 (10.54) |  | 787 (8.52) |  | 3,616 (10.48) |  |  |
| Body mass index (kg/m2) | 27.42 ± 4.78 |  | 27.31 ± 4.90 |  | 27.26 ± 4.77 |  | 0.387 |
| Total cholesterol (mmol/L) | 5.69 ± 1.14 |  | 5.88 ± 1.20 |  | 5.72 ± 1.17 |  | <0.001 |
| HDL-C (mmol/L) | 1.45 ± 0.38 |  | 1.54 ± 0.40 |  | 1.47 ± 0.39 |  | <0.001 |
| LDL-C (mmol/L) | 3.56 ± 0.87 |  | 3.64 ± 0.91 |  | 3.56 ± 0.89 |  | <0.001 |
| Blood pressure |  |  |  |  |  |  |  |
| Systolic blood pressure (mmHg) | 137.72 ± 18.58 |  | 138.27 ± 19.18 |  | 139.35 ± 19.16 |  | <0.001 |
| Diastolic blood pressure (mmHg) | 82.28 ± 10.14 |  | 81.49 ± 10.03 |  | 81.92 ± 10.07 |  | <0.001 |
| Heart rate (bpm) | 69.25 ± 11.22 |  | 72.11 ± 11.42 |  | 69.78 ± 11.40 |  | <0.001 |
| Diabetes, n (%) | 22,432 (5.09) |  | 466 (5.05) |  | 1,933 (5.60) |  | 0.039 |
| Coronary Heart Disease, n (%) | 21,190 (4.81) |  | 317 (3.43) |  | 1,871 (5.43) |  | <0.001 |
| Hypertension, n (%) | 2,273 (0.52) |  | 56 (0.61) |  | 232 (0.67) |  | 0.532 |
| Hypercholesterolaemia, n (%) | 87 (0.02) |  | 4 (0.04) |  | 5 (0.01) |  | 0.191 |
| Chronic Kidney Disease, n (%) | 1,394 (0.32) |  | 62 (0.67) |  | 173 (0.50) |  | 0.057 |
| Obesity, n (%) | 821 (0.18) |  | 10 (0.11) |  | 50 (0.14) |  | 0.492 |
| Medications, n (%) |  |  |  |  |  |  |  |
| Insulin | 4,719 (1.07) |  | 89 (0.96) |  | 393 (1.14) |  | 0.168 |
| Blood pressure medication | 88,971 (20.18) |  | 2,038 (22.08) |  | 8,585 (24.89) |  | <0.001 |
| Cholesterol lowering medication | 74,398 (16.88) |  | 1,433 (15.52) |  | 7,057 (20.46) |  | <0.001 |
| IPAQ (%) |  |  |  |  |  |  | <0.001 |
| Low | 66,953 (15.19) |  | 1,479 (16.02) |  | 5,266 (15.27) |  |  |
| Moderate | 145,073 (32.91) |  | 3,065 (33.20) |  | 11,276 (32.70) |  |  |
| High | 144,526 (32.79) |  | 2,625 (28.43) |  | 10,900 (31.61) |  |  |
| Overall health rating, n (%) |  |  |  |  |  |  | <0.001 |
| Excellent | 74,382 (16.87) |  | 775 (8.39) |  | 4,533 (13.14) |  |  |
| Good | 225,845 (58.04) |  | 5,034 (54.53) |  | 19,249 (55.81) |  |  |
| Fair | 89,779 (20.37) |  | 2,585 (28.00) |  | 8,379 (24.30) |  |  |
| Poor | 18,218 (4.13) |  | 738 (7.99) |  | 2,102 (6.09) |  |  |
| ARIC-HF (%) | 1.12 ± 2.03 |  | 1.01 ± 1.51 |  | 1.46 ± 2.30 |  | <0.001 |
| PCP-HF (%) | 2.23 ± 1.88 |  | 2.47 ± 1.89 |  | 2.88 ± 2.21 |  | <0.001 |
| CORA, n (%) |  |  |  |  |  |  | <0.001 |
| Low | - |  | 5,273 (57.12) |  | 18,129 (52.57) |  |  |
| Medium | - |  | 3,327 (36.04) |  | 13,299 (38.56) |  |  |
| High | - |  | 528 (5.72) |  | 2,663 (7.72) |  |  |
| Very high | - |  | 38 (0.41) |  | 87 (0.25) |  |  |
| Heart Failure incidence, n (%) | 13,059 (2.96) |  | 479 (5.19) |  | 1,491 (4.32) |  | <0.001 |
| Days post consent (days) | 2,887.31 ± 1,258.65 |  | 2,790.83 ± 1,262.88 |  | 2,846.71 ± 1,268.76 |  | 0.401 |

HDL-C: High-density lipoprotein cholesterol; LDL-C: Low-density lipoprotein cholesterol; IPAQ: International Physical Activity Questionnaire; ARIC-HF: Atherosclerosis Risk in Communities – Heart Failure; PCP-HF: Pooled Cohort Equations to Prevent Heart Failure; CORA: Cardio-Oncology Risk Assessment.

Supplementary Table 6: HF incidence among subgroups of participants at different timeline post cancer diagnoses.

|  | Breast cancer and lymphoma | | | *p* |  | Other cancer types | | | *p* |
| --- | --- | --- | --- | --- | --- | --- | --- | --- | --- |
|  | <5 years  (n = 4,091) | between 5-10 years  (n = 3,100) | <10 years  (n = 2,040) |  |  | <5 years  (n = 7,860) | between 5-10 years  (n = 4,904) | <10 years  (n = 11,024) |  |
| HF incidence, n (%) | 208 (5.08) | 159 (5.13) | 112 (5.49) | 0.783 |  | 259 (3.30) | 177 (3.61) | 395 (3.58) | 0.803 |

HF: Heart failure. The duration of cancer diagnosis was defined as the duration between the date of cancer diagnoses to consent date.
